# It is time to reassess reporting of electroconvulsive therapy data in New Zealand: A 17-year retrospective analysis of treatment data from Waikato

**DOI:** 10.1177/00048674251324795

**Published:** 2025-03-17

**Authors:** Robert M Lundin, Savani Kannangara, Matthew Jenkins, Teresa Carroll, Kathrine Wakefield, Colin Patrick, Moloud Abdar, Abbas Khosravi, Colleen Loo, Michael Berk

**Affiliations:** 1 Institute for Mental and Physical Health and Clinical Translation (IMPACT), School of Medicine, Deakin University, Geelong, VIC, Australia; 2 Change to Improve Mental Health (CHIME), Barwon Health MHDAS, University Hospital Geelong, Geelong, VIC, Australia; 3 Peter Rothwell Academic Centre, The University of Auckland, Waikato Hospital, Hamilton, New Zealand; 4 Mental Health and Addiction Services, Te Whatu Ora, Waikato, New Zealand; 5 Institute for Intelligent Systems Research and Innovation (IISRI), Deakin University, Geelong, VIC, Australia; 6 Discipline of Psychiatry and Mental Health, School of Clinical Medicine, NSW, Australia; 7 Black Dog Institute, Sydney, NSW, Australia

**Keywords:** Electroconvulsive therapy, electroconvulsive therapy, New Zealand, neurostimulation

## Abstract

**Objective:**

The New Zealand Government has provided brief annual reports on electroconvulsive therapy treatment since 2004. Despite this, only limited information is made available to clinicians to guide clinical improvement and refine guidelines. Beyond an audit from Otago detailing 10 years of electroconvulsive therapy treatments, limited information is available about electroconvulsive therapy treatments in New Zealand. This paper reports on the use of electroconvulsive therapy over the past 17 years in one New Zealand District Health Board.

**Methods:**

It covers 7126 treatments for 333 patients between 2004 and 2020.

**Results:**

Despite an increasing number of treatments, there has been no per capita growth when corrected for the population. Despite criticism for the disproportionate use of electroconvulsive therapy in women, treatment equity for men and women has been evidenced over the latter 3 years. The majority of treatments were given under voluntary consent, even among patients admitted under the Mental Health Act. Clinical practice is moving towards bifrontal treatment over other electrode placements in response to clinical guidance.

**Conclusion:**

While COVID-19 had broad impacts across healthcare services, it has not led to an overall change in treatments. There was, however, a noticeable shift towards more psychotic disorders treated during the pandemic. This study also provides data that Māori and Pacific Islanders are accessing electroconvulsive therapy, though rates of electroconvulsive therapy usage are still lesser on a proportional population basis. Conclusions are provided to improve national data collection and reporting standards.

## Introduction

Electroconvulsive therapy (ECT) is a treatment for severe mental health conditions that is well-established in New Zealand and is recommended for treatment-resistant depression in treatment guidelines (Weiss et al., 2019). Despite this, the evidence base for ECT has often been overshadowed by public opinion (Clarke, 2019).

A petition was presented in 1999 to the New Zealand Government that requested the practice of ECT to be banned due to its ‘degrading and inhumane nature’. At the time, there were no routine ECT data being recorded on a national scale. With strong dissenting opinions on compulsory treatment under The Mental Health (Compulsory Assessment and Treatment) Act 1992, the House of Representatives recommended a significant change in how ECT is reported (Melding, 2006).

By mid-2002, the Ministry of Health had commissioned a review of the efficacy, safety and regulatory controls surrounding ECT practices in the country. This was published in 2005 alongside the comprehensive 2005 National ECT Audit to provide an understanding of the quality of ECT delivery and establish baseline data for future comparison (Ministry of Health, 2005). From this point onwards, yearly data on the use of ECT was reported as part of the Office of the Director of Mental Health and Addiction Services Annual Report (Ministry of Health, 2021).

The initial 2005 audit acknowledged the benefit and necessity of this treatment for highly selected patients and recommended a continuing audit cycle to ensure continued refinement of the delivery of ECT (Ministry of Health, 2005). However, no further national audits have been completed in the 17 years following.

ECT treatment data reported in the annual reports stratify only a few main categories of population data such as ECT by region, sex and age of patients, ethnicity of patients and status of consent and capacity. These reports neglect to include any analysis of the data or critique of current practice that would allow for secondary analysis (Melding, 2006; Ministry of Health, 2021). No national New Zealand data are, therefore, available to demonstrate the safety or efficacy of treatment.

In 2014, an audit of a single ECT centre in Otago recognised the lack of patient-level data released by the Ministry of Health and the gap in ECT clinical audit that this created. Using 10 years of ECT and clinical file data, a more in-depth analysis was completed. The 2014 audit covered ECT utilisation with medication, number of treatments in a course of ECT and inter-treatment intervals for patients of different diagnoses and treatment responses (Alvarez-Grandi et al., 2014). This patient-level data taken for over a decade was integral to the findings presented but no further audits in New Zealand have attempted to replicate this analysis.

ECT practices around the country vary greatly in both utilisation of services and the way these services are provided (Melding, 2006). The most recent survey of ECT practice in New Zealand took place in 2015 and surveyed all 16 providers of ECT at the time. It highlighted that New Zealand had a lower utilisation of ECT than Australia and primarily used unilateral and bitemporal treatment, using propofol for induction of general anaesthetic. It also highlighted a lack of dedicated facilities and allocation of clinical time for ECT psychiatrists (Fisher et al., 2017). This variation is attributed to the lack of a single protocol for ECT administration in New Zealand. Instead, the Royal Australian and New Zealand College of Psychiatrists (RANZCP) professional practice guidelines outline the various evidence-based techniques of administration. Much of the RANZCP guidelines and stance on ECT emphasise the need for evidence-based practice and advocacy to manage the explicit stigma towards ECT and ensure that the most unwell patients who need the therapy are able to receive it (Royal College of Psychiatrists, 2017; Weiss et al., 2019).

These binational RANZCP guidelines, in addition to recommending specific data to be collected, states that an annual clinical audit process should take place but refers directly to ‘national guidelines’ for treatment protocols, documentation and legislation. The recent development of an Australian Clinical Alliance and Research in ECT (CARE) Network has highlighted the potential impact of pooled ECT data by large consortiums in answering key clinical gaps in the literature. Recommendations include standardised recording of treatment outcomes and side effects with the relevant health authority considering clinical changes to address the findings (Clarke, 2019; Dong et al., 2024; Martin et al., 2018). It has also been shown that routine ECT data can be combined with genetics and imaging studies to make meaningful improvements in care (Leaver et al., 2018; Soda et al., 2020).

Moving from multiple district health boards to a single Health New Zealand authority (previously Te Whatu Ora) provides a unique opportunity to work towards a unified national approach to treatment protocols and data collection for ECT in New Zealand (New Zealand Government, 2021). Should there be a new movement against ECT in New Zealand, mimicking the concerns raised in the early 1990s, there is a risk that patients might have reduced access to a treatment that is effective and often used when no other options have succeeded. Reduced use is increasingly possible with the emergence of other neurostimulation treatments, such as transcranial magnetic stimulation, which randomised controlled trials (RCTs) have found to be less effective in more severe forms of depression but have a more tolerable side-effect profile and less stigma attached (Berlim et al., 2013). The use of ketamine has also evolved as an alternative for treatment-resistant depression, which has shown high efficacy in RCTs (Nikolin et al., 2023), in addition to a rapid rise in psychedelics research (Berkovitch et al., 2021). These issues highlight the need for high-quality ECT research, including large-scale consortiums using new technology such as machine learning algorithms (Lundin and Menkes, 2021a, 2021b).

Considering the limited literature on the practice of ECT in New Zealand and the emergence of new treatments for depression, it is crucial to obtain up-to-date data on current practice and statistics. This article presents the largest known patient-level clinical dataset of ECT treatments in New Zealand over the past 17 years through data from the Waikato region. The region covers a population of roughly 500,000 people, immediately south of Auckland on the north island of New Zealand (Statistics New Zealand, 2022). The region’s only ECT service is delivered at Waikato Hospital in Hamilton, servicing a 21,000 km^2^ region (Health New Zealand, 2022). The region is unique is having a large rural population (41%). We specifically focused on capturing changes in ECT treatment and patient demographics over time in addition to trends in compulsory treatment. This paper aims to identify key treatment data that will allow for meaningful comparisons across New Zealand in the future. Finally, we identify possible future work and discuss the need for Health New Zealand (Te Whatu Ora) to implement a more detailed annual report and standardised data collection.

## Methods

ECT treatments at Waikato Hospital between the period from 1 July 2004 to 30 June 2021 were recorded in handwritten logbooks by the ECT treatment team. All treatments had been carried out using the Thymatron® System IV (SOMATICS, LLC, Florida, USA). These records contained the details of treatment, including a national health identifier (NHI), treatment number (within a course of ECT), legal status (consented or treated under section 60B of the Mental Health Act (MHA)), inpatient/outpatient status and diagnoses. Also included in the logbook were details of the ECT treatment, including the charge delivered (recorded as percentage of the device output, 100% = 504 mC), electrode placement, length of the motor seizure, length of electroencephalogram (EEG) recorded seizure, and the type and dose of general anaesthetic and muscle relaxants used.

The paper logbooks were transcribed into an excel spreadsheet, and the treatment records were deidentified using study IDs. Names of patients, treating doctors, anaesthetists and nurses involved were omitted from the data transcription. Key demographics for each patient covering age, sex and ethnicity were obtained from hospital electronic patient records before anonymization.

Multiple ECT stimulations in a single treatment session were recorded as separate individual entries for consistency and given the same patient and treatment characteristics. For the analysis, the final threshold stimulus and parameters were recorded as the treatment session. Where multiple diagnoses were listed for the same patient during a treatment course, the diagnosis recorded for the majority of treatments was used in the analysis. The exception to this was the presence of catatonia or NMS, which was recorded for only the treatment where the feature was noted. This paper differentiates between a stimulation (a single dose of ECT which may or may not lead to a seizure), a treatment (one or more stimulations given in subsequent order to induce a seizure) or a course (a series of treatments). Where treatments were provided two or three times per week the course was considered as acute, and if given once per week or less frequently, then it was considered as maintenance. It was also noted for each treatment if the patient was receiving ECT under own consent or that of a substitute decision-maker. This was irrespective of the patient being treated under the MHA as patients, if deemed to have capacity, can still consent to ECT treatment while under the Act.

To align with the data reported by the New Zealand government, a year is defined as the period from 1 July to 30 June of the following calendar year. Estimated population values were provided by the New Zealand Government’s public datasets (Statistics New Zealand, 2022). The project was registered with the Waikato District Health Board Clinical Audit Support Unit (CASU) with registration #4207.

All statistical analysis were carried out using IBM SPSS version 29 for the Macintosh operating system (IBM Corp, 2022). We used descriptive statistics to describe the demographic characteristics of the participants who received ECT treatment at the Waikato site. A comparison of the demographic characteristics and treatment data for each year between 2004 and 2020 was performed using Pearson’s chi-squared test of independence. Significant differences were determined by *p* values < 0.05.

## Results

### Patient demographics and treatment data

The Waikato dataset represents 7126 individual treatments between July 2004 and July 2021 for 333 patients. There were 742 courses of ECT initiated, with each course averaging 9.6 treatments with a predominantly female patient group (58.6%) over the period investigated. Most patients treated were of New Zealand European (Caucasian) ethnicity, and 15.3% of patients were Māori or Pacific Islander. The remaining ethnicities were recorded as Other European (27), Indian (5), Chinese (1) or unknown (16). Table 1 outlines the characteristics of patients.

**Table 1.** Summary of patient characteristics.

*Characteristic*	Sub-group	n (%)
*Gender*	Female	195 (58.6)
	Male	138 (41.4)
*Ethnicity*	NZ European	233 (70)
	Māori and Pacific Islander	51 (15.3)
	Other	49 (14.7)
*Diagnosis*	Depressive disorders	5840 (82.0)
	Psychotic disorders	788 (11.1)
	Bipolar affective disorder	421 (5.9)
	Anxiety disorders	77 (1.1)
*Age (at first recorded treatment)*	Average age	53.7 (18-91)
	<20	2 (0.6)
	20–29	40 (12.0)
	30–39	37 (11.1)
	40–49	51 (15.3)
	50–59	68 (20.4)
	60–69	67 (20.1)
	70–79	44 (13.2)
	80–89	22 (6.6)
	90+	2 (0.6)

The primary diagnosis recorded for those receiving ECT was a depressive disorder (82.0%) and included treatments for depression (*n* = 5775) and post-partum depression (*n* = 65). Psychotic disorders made up 11% of treatments and include schizophrenia (*n* = 331), depression with psychosis (*n* = 191), schizoaffective disorder (*n* = 146) and psychosis not otherwise specified (*n* = 119). Bipolar affective disorder (BPAD) made up 6% of treatments and covered both manic (*n* = 49) and depressive episodes (*n* = 372). Finally, anxiety disorder (*n* = 77) was the primary diagnosis recorded for 1.1% of patients. However, no subcategories were recorded for anxiety.

Most courses were initiated during inpatient care (519, 69.5%), with 61.5% of all treatments provided during a hospital admission. A summary of the treatment characteristics is presented in Table 2. Although almost half of the courses were started while the patient received care under the MHA (*n* = 334, 45%), only 27.7% of courses were started with substituted consent due to temporary lack of capacity. With legal status frequently changing during a course, 76.6% of all treatments delivered were accepted voluntarily by the patients. Treatments were primarily delivered with bitemporal lead placement (65.2%) compared with other placement types. Propofol was the most common anaesthetic induction agent used in 5402 treatments (75.8%).

**Table 2.** Treatment characteristics.

	Variable	*n* (%)
*Treatments*	Stimulations	8445
	Treatments	7126
	Courses	742
*Admission status*	Inpatient	4383 (61.5)
	Outpatient	2743 (38.5)
*Legal status*	Compulsory	1975 (27.7)
	Voluntary	5151 (72.3)
	Under MHA	1170 (22.7)
*Lead placement*	Bitemporal	4649 (65.2)
	Bifrontal	589 (8.3)
	Right Unilateral	1855 (26.0)
	Left Unilateral	33 (0.5)
*Anaesthetic induction agent*	Propofol only	5402 (75.8)
	Thiopentone only	1239 (17.4)
	Ketamine only	48 (0.7)
	Combination	437 (6.1)
	Ketamine and Propofol	434 (99.1)

### Changes in treatment data and patient numbers

**Figure 1.**
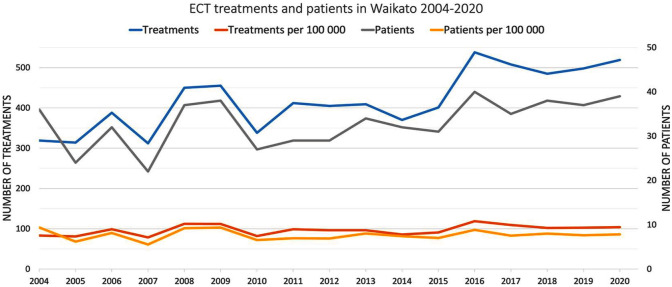
Trends in the number of ECT treatments (left axis) and patients (right axis), including adjustment for population growth in the Waikato region as presented per 100,000 of the Waikato population per year.

The longitudinal changes in treatment data and patient numbers are shown in Figure 1, with annual figures reported in Supplemental Material Appendix 1. Despite an overall increase of 200 treatments from 319 in 2004 to 519 in 2020 (*r* = 0.785, *p* =< 0.001), the growth is not seen when corrected for the population growth of the Waikato region during this period (*r*^2^ = 0.218, *p* = 0.068). A similar development is seen in the number of patients treated, with an increase of three patients per year from 2004 to 2020 when corrected for population growth (*r*^2^ = 0.000, *p* = 0.973).

### Changes to patient demographics

**Figure 2.**
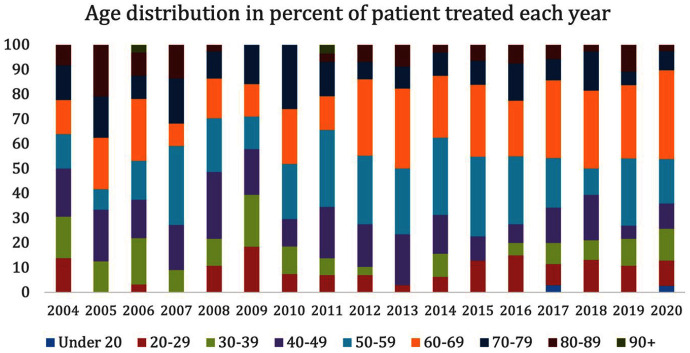
Age distribution of patients receiving treatment for each year between 2004 and 2020.

The distribution of age groups treated in the Waikato dataset had higher variability between 2004 and 2009 (Figure 2). However, most patients treated were younger than 60 years old throughout the period, except for the year 2005. The age group 50–70 became the largest population group in 2012 and remained the highest proportion, except for 2018.

**Figure 3.**
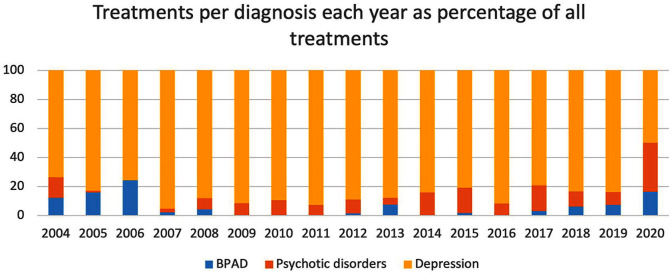
Number of treatments provided for each diagnosis per year between 2004 and 2020.

The frequency of treatments for each diagnosis is listed in Figure 3. Throughout the reporting period, depression remained the most treated disorder. However, a shift was seen from 2019 to 2020, where an increase in treatment of psychotic (300%) and BPAD (130%) was seen. As there was only a minimal difference in the total number of treatments (498 vs 519 treatments) from 2019 to 2020, a corresponding decrease was observed in the number of depression (34%) treatments. An isolated spike in the treatment of those with anxiety disorders was seen in 2019, and yearly incremental increases in the treatment of those with bipolar disorder from 2004 to 2006 and again from 2017 to 2020.

The main diagnosis at the time of ECT treatment remained consistent for 308 patients without any change over time. Twenty-five patients had received courses of ECT at different times for differing diagnoses, where the most common combination was psychosis and depression (*n* = 14). Patients also received treatment for (unipolar) depression and mania (*n* = 8), depression and anxiety (*n* = 2) and psychosis and mania (*n* = 1). There were 27 patients treated for catatonia over 140 treatments, with an average of 5 treatments per patient. Of these, the main diagnoses were depression (59%), psychotic disorders (30.7%) and BPAD (1.0%).

**Figure 4.**
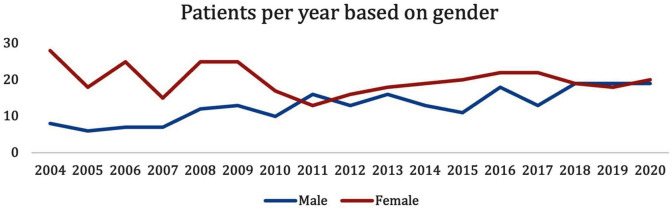
Number of patients by gender from 2004 to 2020.

Although the overall dataset predominantly comprised female patients (58.6%), there was a steady increase of treatments in male patients (Figure 4). This led to more male than female patients treated in 2011 and treatment ratios close to 50% over the past 3 years (Figure 4).

**Figure 5.**
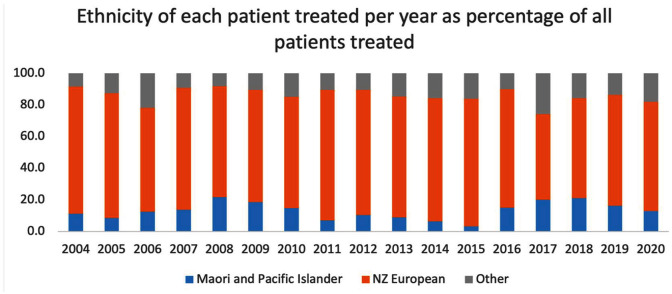
Ethnicity of each patient per year between 2004 and 2020 as a percentage of all patients treated.

Māori and Pacific Islander patients made up 15.3% of all patients treated but received a smaller proportion of the total treatments at 12.1% (Figure 5). They were also more likely to be placed under the MHA (55.7%) than the other patients (42.5%) and received a higher proportion of involuntary treatments (31.8% vs 27.1%).

### Changes to treatment characteristics

**Figure 6.**
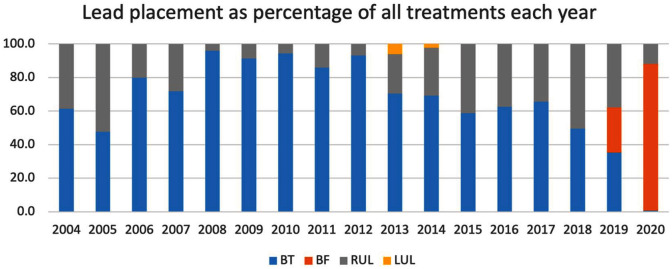
Distribution of ECT lead placements for each treatment per year between 2004 and 2020 as percentage of all treatments provided.

The data highlight a shift from providing primarily bitemporal placement of ECT leads from 2004 to bifrontal in 2019. Bifrontal lead placement became the most common placement from 2020 onwards. Patients only received right unilateral treatment where it was clinically indicated above bilateral options and only a small proportion of patients received left unilateral treatment (Figure 6).

**Figure 7.**
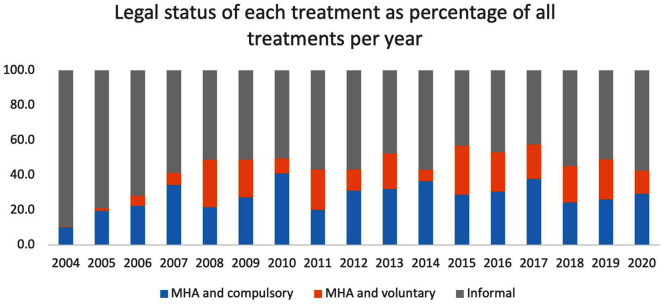
Number of treatments provided for each diagnosis per year between 2004 and 2020.

An increase in the proportion of ECT given under the MHA was seen in Waikato since 2004 (Figure 7). Overall, 3145 (44.1%) of treatments were delivered while the patient was under the MHA, but only 27.7% of these were delivered under the Act because a patient lacked capacity.

## Discussion

Overall, the data presented correspond closely to the clinical audit from Otago and the New Zealand national report. However, differences in data collection methods and overlap with the data gathered in the national data make direct comparisons inexact. The focus of this article was therefore reporting of the Waikato dataset as opposed to meaningful comparisons. It is also important to keep in mind that the Waikato and Otago data are included within the national dataset referenced. It is nonetheless helpful to point out that treatments in Waikato were above the 2020 New Zealand national average of 4.6 patients per 100,000 of the population where it provided 16.8% of ECT patients and 19.7% of treatments in New Zealand despite holding only 10% of the national population. The data are also consistent with the New Zealand government report where the average number of treatments per patient sits at 11.4 while the Waikato average treatments per course were slightly lower at 9.6 treatments.

The relative stability seen in Waikato treatment numbers over time possibly reflects a saturation of resources available for treatment or minimal change in the need for treatment. Although the number of treatments per patient changed over time, it is difficult to assess whether this was associated with clinical need or service availability without more detailed statistics. This possibly also reflects that changes to guidelines and treatment procedures do not influence access to treatment overall. When planning service delivery, this might indicate that aiming for a minimum of 8 patients and/or 100 treatments per 100,000 people is necessary for comparable health services.

Despite differences in treatment numbers, there appears to be more consistency in the presenting diagnoses. Treatments in Otago were primarily given to those with depressive disorders, BPAD and psychotic disorders, making up 82, 5.9 and 11.1% of the patient group respectively compared with 74.8, 14.0 and 12.2% in Waikato. These figures remain comparable to previously reported combined figures for Australia and New Zealand (66–82%, 5–9% and 2–29%) with a similar diagnosis distribution to the United States (72–92% affective disorders and 8–29% psychotic disorders) and European countries (62–89%, 1–9% and 5–29%). By comparison, most African, South American and Asian countries (at 60–83%, 50–55% and 30–80%) typically report the opposite pattern of majority psychotic disorders followed by affective disorders, with notable exceptions to the above regional patterns being Turkey, Russia, Hungary, Pakistan, Saudia Arabia and Hong Kong (Leiknes et al., 2012).

Interestingly, there was no drop in the number of treatments during the early stages of the COVID-19 period that our data cover. This indicates that ECT is a crucial service that needs to have emergency contingencies. There was, however, a noticeable shift in the diagnoses treated towards psychosis and bipolar disorder at the expense of depression, possibly reflecting relapses due to increased psychosocial stressors, changes to treatment triaging and reduced contact with community services. Future work should attempt to correlate these findings to inpatient admissions to see if the patterns correlate across New Zealand. Although there was initially some variability in the age groups treated, this appeared to stabilise from 2009 onwards. It remains unclear if this was due to local treatment practices or wider population changes.

The female-to-male ratio for the full dataset was lower at 58.6% women in Waikato compared with 67.3% in Otago. However, when assessed over time it becomes apparent that Waikato has reached equivalence in male and female treatments over the past 3 years of the data. It is important to consider possible reasons for this as a higher rate for women is often framed as overtreatment, but the alternative interpretation is that men are often undertreated. Rates of treatment should be interpreted in light of data that women present with depression at a higher rate than men, but the prevalence of severe and treatment-resistant depression is closer to gender parity (Østergaard et al., 2023). If this trend continues in Waikato, then it would be important to identify the factors that might have led to the change in treatment demographic as no active work has been undertaken to address gender differences. For example, with the advent of alternative treatments, the use of ECT may have shifted to the treatment of more severe and treatment-resistant depression, thus reflecting more parity in the rates of ECT treatment in men and women in recent years. It also highlights why looking at trends over time can provide crucial insights over the yearly reported averages from the New Zealand dataset.

Data on ethnicities will also reflect that Waikato has a Māori and Pacific Islander population higher than the national average at 27 vs 23.3% nationally. While the Otago patients were predominantly NZ European, the Waikato group includes 15.3% Māori and Pacific Islander people and thus is higher than the current national ECT treatment average of 12.9% in 2020. This likely represents a well-recognised health equity and socioeconomic issues regarding access to mental health treatments.

The observed changes in treatment lead placement demonstrates, in Figure 6, a very rapid change from bitemporal to bifrontal lead placement as the default lead placement for bilateral treatments in 2019 (from 1/7/2019 to 30/6/2020). This change in practice may be related to the publication of the binational RANZCP guidelines in April 2019, which noted the higher risk of retrograde amnesia with BT ECT, suggesting it is not used as an initial treatment option but reserved for situations where other forms of ECT are not appropriate. This information was also disseminated to clinicians through training courses in Australia and New Zealand. If so, this demonstrates that change can occur over a short period of time in response to the release of professional practice guidelines assisted by dissemination of information to clinicians through training courses.

For unilateral ECT, a high variability in use of unilateral treatment options was seen. The data show a reduction in use of RUL ECT between 2004 and 2008, increase over 2011–2018, then decrease in 2019–2020. These trends may have been driven by uptake of ultrabrief RUL ECT after 2008, with the emergence of evidence of efficacy and superior cognitive outcomes from clinical trials (Sackeim et al., 2008; Tor et al., 2015). Our dataset is unable to confirm if this was the reason, as pulsewidth (brief vs ultrabrief) was not consistently coded. The decline in use of RUL ECT in 2019–2020 occurred in the context of Covid, and may have been influenced by published opinions recommending that highly effective forms of ECT be used during the Covid epidemic, to ensure a rapid response with the minimal number of treatments (Kwan et al., 2022).

We have speculated on external factors (practice guidelines, Covid epidemic) that may have influenced ECT prescribing but do not necessarily explain variability between individual clinical centres. This further makes the argument for national benchmarking that allow regional sites to annually appraise their own practice against those of other regions and services, and for clinicians to keep abreast of current recommendations for practice (Gill et al., 2023).

It is more difficult to compare legal data on involuntary treatments as the New Zealand government only highlights the number of patients treated involuntarily (compulsory) but does not include patients who are under MHA and consenting to treatment as these legal frameworks are separate. This is a strong argument for reconsidering the nature of the data collected and published by health services which would also allow comparisons between regions and to other countries where similar data are reported (Krarup et al., 2024; Livingston et al., 2018).

### Limitations

There are significant limitations associated with the dataset provided in this paper, primarily the lack of data on clinical improvement, side effects and medications used at the time of treatment. It also represents ECT data from the only ECT service in the Waikato region. As such, there will be local policies and practices that mean the findings cannot be generalised to all of New Zealand. The data were also retrospectively collected from paper notes where a degree of transcription error and illegible entries are unavoidable, and there is no way to control for missing data. Furthermore, diagnostic information was limited to the information provided at the time of referral. The current dataset does not differentiate between brief and ultrabrief right unilateral treatment which means that both categories have unfortunately been grouped.

The data presented provide several opportunities for secondary analysis of the legal framework, use of anaesthetic medications and their impact, relapse rates and patient factors such as treatment quality, gender and ethnicity. If these data are going to be collected on a national scale with modernised collection tools, ideally within electronic health records, then the large quality dataset combined with known literature gaps could be analysed using machine learning studies (Lundin et al., 2024).

## Conclusion

This paper provides a detailed overview of ECT treatments in the Waikato region over the past 17 years. Although cursory data is reported on a national scale every year, health services should carefully consider how they collect and report data to provide more meaningful datasets for modern mental health services. The CARE dataset is one such example (Martin et al., 2018). The change from individual District Health Boards to Health New Zealand provides a solid foundation for this work to be undertaken, particularly if clinical data can be collected using a national database to allow joint analysis. Over time, the gathered data should inform treatment guidelines for New Zealand.

We, therefore, make the argument that data collected by the government should include key details on the treatment parameters such as those captured by the CARE framework (Martin et al., 2018). Using an existing framework would avoid duplication of work and allow direct comparisons between ECT in New Zealand and other partners in the Australasian region. It would also be sensible to collect data on voluntary treatments under the MHA to show how the patient’s journey moves from compulsory treatment to one where the patient recognises its importance. Data on medications used and lead placements would also help inform discrepancies between sites. From a health equity perspective, it becomes important to identify whether similar increases in access for male patients are seen at other sites. Reduced access to ECT for Māori and Pacific Islander populations is a significant concern that needs to be addressed alongside known barriers to access to mental healthcare. Very little is known about relapse rates in New Zealand, and this needs to be clearly outlined alongside any use of maintenance ECT. Many of these factors would move to address the significant stigma and critique of ECT. Gathering data on experienced side effects and clinical efficacy using standardised tools would further support this issue.

## Supplemental Material

sj-docx-1-anp-10.1177_00048674251324795 – Supplemental material for It is time to reassess reporting of electroconvulsive therapy data in New Zealand: A 17-year retrospective analysis of treatment data from Waikato Supplemental material, sj-docx-1-anp-10.1177_00048674251324795 for It is time to reassess reporting of electroconvulsive therapy data in New Zealand: A 17-year retrospective analysis of treatment data from Waikato by Robert M Lundin, Savani Kannangara, Matthew Jenkins, Teresa Carroll, Kathrine Wakefield, Colin Patrick, Moloud Abdar, Abbas Khosravi, Colleen Loo and Michael Berk in Australian & New Zealand Journal of Psychiatry

## References

[bibr1-00048674251324795] Alvarez-Grandi S delaBarra SL Seifert A , et al. (2014) Electroconvulsive therapy use in Otago, New Zealand: A 10-year retrospective audit of patient-level treatment data. Australian and New Zealand Journal of Psychiatry 48: 548–553. 24253357 10.1177/0004867413514119

[bibr2-00048674251324795] Berkovitch L Roméo B Karila L , et al. (2021) Efficacy of psychedelics in psychiatry, a systematic review of the literature. L’Encephale 47: 376–387. 10.1016/j.encep.2020.12.002 33888297

[bibr3-00048674251324795] Berlim MT VandenEynde F Daskalakis ZJ (2013) Efficacy and acceptability of high frequency repetitive transcranial magnetic stimulation (rTMS) versus electroconvulsive therapy (ECT) for major depression: A systematic review and meta-analysis of randomized trials. Depression and Anxiety 30: 614–623. 23349112 10.1002/da.22060

[bibr4-00048674251324795] Clarke P (2019) Hip hip hooray, ECT turns 80! Australasian Psychiatry 27: 53–55. 10.1177/1039856218815753 30474389

[bibr5-00048674251324795] Dong V Brettell L Massaneda-Tuneu C , et al. (2024) Facilitating routine data collection to improve clinical quality and research in Interventional Psychiatry: The CARE Network. Australian and New Zealand Journal of Psychiatry 58: 738–741. 39054785 10.1177/00048674241266057 PMC11370177

[bibr6-00048674251324795] Fisher MW Morrison J Jones PA (2017) Electroconvulsive therapy practice in New Zealand. The Journal of ECT 33: 134–137. 27922459 10.1097/YCT.0000000000000364

[bibr7-00048674251324795] Gill S Hussain S Purushothaman S , et al. (2023) Prescribing electroconvulsive therapy for depression: Not as simple as it used to be. Australian and New Zealand Journal of Psychiatry 57: 1202–1207. 37353902 10.1177/00048674231183368

[bibr8-00048674251324795] Health New Zealand (2022) Snapshot of Health New Zealand – waikato. Snapshot of Health New Zealand – Waikato” Te Whatu Ora Health New Zealand Waikato. Available at: www.waikatodhb.health.nz/about-us/snapshot-of-waikato-dhb/ (accessed 19 December 2024).

[bibr9-00048674251324795] IBM Corp Released (2021) IBM SPSS Statistics for Windows, Version 29.0. Armonk, NY: IBM Corp.

[bibr10-00048674251324795] Krarup M Kellner CH Østergaard SD (2024) Clinical and legal differences in the use of involuntary electroconvulsive therapy for life-threatening illness across European countries. The Journal of ECT 40: 105–110. 38194602 10.1097/YCT.0000000000000984

[bibr11-00048674251324795] Kwan E Le B Loo CK , et al. (2022) The Impact of COVID-19 on electroconvulsive therapy: A multisite, retrospective study from the clinical alliance and research in electroconvulsive therapy and related treatments network. The Journal of ECT 38: 45–51. 34387286 10.1097/YCT.0000000000000800 PMC8875438

[bibr12-00048674251324795] Leaver AM Wade B Vasavada M , et al. (2018) Fronto-temporal connectivity predicts ECT outcome in major depression. Frontiers in Psychiatry 9: 92. 29618992 10.3389/fpsyt.2018.00092 PMC5871748

[bibr13-00048674251324795] Leiknes KA Jarosh-vonSchweder L Høie B (2012) Contemporary use and practice of electroconvulsive therapy worldwide. Brain and Behavior 2: 283–344. 22741102 10.1002/brb3.37 PMC3381633

[bibr14-00048674251324795] Livingston R Wu C Mu K , et al. (2018) Regulation of electroconvulsive therapy: A systematic review of US state laws. The Journal of ECT 34: 60–68. 28991068 10.1097/YCT.0000000000000460

[bibr15-00048674251324795] Lundin RM Menkes DB (2021a) Commentary: Managing virtual hybrid psychiatrist-patient relationships in a digital world. Frontiers in Public Health 9: 664778. 33928066 10.3389/fpubh.2021.664778 PMC8076494

[bibr16-00048674251324795] Lundin RM Menkes DB (2021b) Realising the potential of digital psychiatry. The Lancet Psychiatry 8: 655. 34174199 10.1016/S2215-0366(21)00165-6

[bibr17-00048674251324795] Lundin RM Falcao VP Kannangara S , et al. (2024) Machine learning in electroconvulsive therapy: A systematic review. The Journal of ECT 40: 245–253. 38857315 10.1097/YCT.0000000000001009

[bibr18-00048674251324795] Martin DM Gálvez V Lauf S , et al. (2018) The clinical alliance and research in electroconvulsive therapy network: An Australian initiative for improving service delivery of electroconvulsive therapy. The Journal of ECT 34: 7–13. 28658011 10.1097/YCT.0000000000000435

[bibr19-00048674251324795] Melding P (2006) Electroconvulsive therapy in New Zealand: Terrifying or electrifying? The New Zealand Medical Journal 119: 1237. 16862197

[bibr20-00048674251324795] Ministry of Health (2005) Electroconvulsive therapy audit report. Available at: www.moh.govt.nz (accessed 15 September 2004).

[bibr21-00048674251324795] Ministry of Health (2021) Office of the Director of Mental Health and Addiction Services: Annual Report 2018 and 2019. Wellington, New Zealand: Ministry of Health.

[bibr22-00048674251324795] New Zealand Government (2021) Our Health and Disability System: Building a Stronger Health and Disability System that Delivers for All New Zealanders’. Wellington, New Zealand: New Zealand Government.

[bibr23-00048674251324795] Nikolin S Rodgers A Schwaab A , et al. (2023) Ketamine for the treatment of major depression: A systematic review and meta-analysis. EClinicalMedicine 62: 102127. 37593223 10.1016/j.eclinm.2023.102127 PMC10430179

[bibr24-00048674251324795] Østergaard SD Seidler Z Rice S (2023) The ICD-11 opens the door for overdue improved identification of depression in men. World Psychiatry 22: 480–481. 37713575 10.1002/wps.21124 PMC10503896

[bibr25-00048674251324795] Royal College of Psychiatrists (2017) Statement on Electroconvulsive Therapy (ECT). Available at: www.rcpsych.ac.uk/docs/default-source/about-us/who-we-are/electroconvulsive-therapy—ect-ctee-statement-feb17.pdf?sfvrsn=2f4a94f9_2 (accessed 15 September 2004).

[bibr26-00048674251324795] Sackeim HA Prudic J Nobler MS , et al. (2008) Effects of pulse width and electrode placement on the efficacy and cognitive effects of electroconvulsive therapy. Brain Stimulation 1: 71–83. 19756236 10.1016/j.brs.2008.03.001 PMC2742986

[bibr27-00048674251324795] Soda T McLoughlin DM Clark SR , et al. (2020) International consortium on the genetics of electroconvulsive therapy and severe depressive disorders (Gen-ECT-ic). European Archives of Psychiatry and Clinical Neuroscience 270: 921–932. 31802253 10.1007/s00406-019-01087-w PMC7385979

[bibr28-00048674251324795] Statistics New Zealand (2022) Infoshare – Population estimation for Waikato. Available at: https://infoshare.stats.govt.nz/ (accessed 15 September 2004).

[bibr29-00048674251324795] Tor PC Bautovich A Wang MJ , et al. (2015) A systematic review and meta-analysis of brief versus ultrabrief right unilateral electroconvulsive therapy for depression. The Journal of Clinical Psychiatry 76: e1092–e1098. 10.4088/JCP.14r09145 26213985

[bibr30-00048674251324795] Weiss A Hussain S Ng B , et al. (2019) ‘Royal Australian and New Zealand College of Psychiatrists professional practice guidelines for the administration of electroconvulsive therapy’. Available at: 10.1177/0004867419839139 30966782

