# Supplementary material for: It is time to reassess reporting of electroconvulsive therapy data in New Zealand: A 17-year retrospective analysis of treatment data from Waikato
Source: Aust N Z J Psychiatry. 2025 Mar 17;59(5):423–32. doi: 10.1177/00048674251324795 (PMC12022368; doi:10.1177/00048674251324795)
Supplement: sj-docx-1-anp-10.1177_00048674251324795 – Supplemental material for It is time to reassess reporting of electroconvulsive therapy data in New Zealand: A 17-year retrospective analysis of treatment data from Waikato [file sj-docx-1-anp-10.1177_00048674251324795.docx]

Appendix 1 – All treatment data from 2004 – 2020

| **Year** |  | **2004** | **2005** | **2006** | **2007** | **2008** | **2009** | **2010** | **2011** | **2012** | **2013** | **2014** | **2015** | **2016** | **2017** | **2018** | **2019** | **2020** |
| --- | --- | --- | --- | --- | --- | --- | --- | --- | --- | --- | --- | --- | --- | --- | --- | --- | --- | --- |
| **Treatments** | Treatments | 319 | 314 | 388 | 312 | 450 | 455 | 338 | 412 | 405 | 409 | 370 | 401 | 538 | 508 | 485 | 498 | 519 |
|  | per 100 000 | 83.0 | 80.8 | 98.7 | 78.5 | 112.1 | 111.9 | 82.0 | 98.7 | 96.1 | 96.3 | 85.6 | 90.7 | 118.8 | 109.2 | 102.0 | 102.5 | 103.9 |
|  | Patients | 36 | 24 | 32 | 22 | 37 | 38 | 27 | 29 | 29 | 34 | 32 | 31 | 40 | 35 | 38 | 37 | 39 |
|  | per 100 000 | 9.4 | 6.2 | 8.1 | 5.5 | 9.2 | 9.3 | 6.5 | 6.9 | 6.9 | 8.0 | 7.4 | 7.0 | 8.8 | 7.5 | 8.0 | 7.6 | 7.8 |
| **gender** | Male | 8 | 6 | 7 | 7 | 12 | 13 | 10 | 16 | 13 | 16 | 13 | 11 | 18 | 13 | 19 | 19 | 19 |
|  | Female | 28 | 18 | 25 | 15 | 25 | 25 | 17 | 13 | 16 | 18 | 19 | 20 | 22 | 22 | 19 | 18 | 20 |
| **Ethnicity** | Māori and Pacific Islander | 4 | 2 | 4 | 3 | 8 | 7 | 4 | 2 | 3 | 3 | 2 | 1 | 6 | 7 | 8 | 6 | 5 |
|  | NZ European | 29 | 19 | 21 | 17 | 26 | 27 | 19 | 24 | 23 | 26 | 25 | 25 | 30 | 19 | 24 | 26 | 27 |
|  | Other | 3 | 3 | 7 | 2 | 3 | 4 | 4 | 3 | 3 | 5 | 5 | 5 | 4 | 9 | 6 | 5 | 7 |
| **Diagnoses** | BPAD | 39 | 50 | 94 | 7 | 19 | 0 | 0 | 0 | 6 | 31 | 0 | 7 | 0 | 16 | 30 | 37 | 85 |
|  | Psychotic disorders | 45 | 4 | 0 | 8 | 35 | 39 | 36 | 30 | 39 | 19 | 59 | 70 | 44 | 90 | 51 | 44 | 175 |
|  | Depression | 235 | 260 | 294 | 297 | 396 | 416 | 302 | 382 | 360 | 359 | 311 | 324 | 494 | 403 | 408 | 417 | 259 |
| **Quality** | Average Seizure | 42.63 | 35.66 | 30.73 | 33.26 | 30.40 | 33.02 | 32.87 | 32.03 | 45.10 | 44.19 | 45.75 | 47.37 | 48.19 | 41.29 | 41.57 | 43.33 | 46.38 |
|  | Median Power | 40 | 50 | 50 | 50 | 50 | 50 | 50 | 50 | 35 | 35 | 35 | 35 | 35 | 50 | 50 | 50 | 35 |
| **Placement** | BT | 196 | 150 | 310 | 224 | 432 | 416 | 319 | 354 | 377 | 288 | 256 | 236 | 337 | 334 | 242 | 175 | 3 |
|  | BF | 0 | 0 | 0 | 0 | 0 | 0 | 0 | 0 | 0 | 0 | 0 | 0 | 0 | 0 | 0 | 135 | 454 |
|  | RUL | 123 | 164 | 78 | 88 | 18 | 39 | 19 | 58 | 28 | 96 | 106 | 165 | 201 | 175 | 247 | 188 | 62 |
|  | LUL | 0 | 0 | 0 | 0 | 0 | 0 | 0 | 0 | 0 | 25 | 8 | 0 | 0 | 0 | 0 | 0 | 0 |
| **Medications** | Propofol | N/A | N/A | 117.8 | 116.0 | 126.0 | 118.3 | 117.8 | 125.8 | 67.7 | 61.1 | 66.0 | 70.0 | 67.6 | 62.0 | 64.4 | 68.4 | 69.8 |
|  | Thiopentone | 240.2 | 249.2 | 218.4 | 286.4 | 287.4 | 252.7 | 241.6 | 270.3 | N/A | N/A | N/A | N/A | N/A | N/A | N/A | N/A | N/A |
|  | Ketamine | N/A | N/A | N/A | N/A | N/A | N/A | N/A | N/A | 50.0 | 44.1 | 40.5 | 41.7 | 46.3 | 40.5 | 34.3 | 46.0 | 41.8 |

| **Ages** | **Average age** | **52** | **62** | **56** | **59** | **51** | **48** | **57** | **56** | **56** | **59** | **54** | **56** | **55** | **55** | **55** | **56** | **54** |
| --- | --- | --- | --- | --- | --- | --- | --- | --- | --- | --- | --- | --- | --- | --- | --- | --- | --- | --- |
|  | Under 20 | 0 | 0 | 0 | 0 | 0 | 0 | 0 | 0 | 0 | 0 | 0 | 0 | 0 | 1 | 0 | 0 | 1 |
|  | 20-29 | 5 | 0 | 1 | 0 | 4 | 7 | 2 | 2 | 2 | 1 | 2 | 4 | 6 | 3 | 5 | 4 | 4 |
|  | 30-39 | 6 | 3 | 6 | 2 | 4 | 8 | 3 | 2 | 1 | 0 | 3 | 0 | 2 | 3 | 3 | 4 | 5 |
|  | 40-49 | 7 | 5 | 5 | 4 | 10 | 7 | 3 | 6 | 5 | 7 | 5 | 3 | 3 | 5 | 7 | 2 | 4 |
|  | 50-59 | 5 | 2 | 5 | 7 | 8 | 5 | 6 | 9 | 8 | 9 | 10 | 10 | 11 | 7 | 4 | 10 | 7 |
|  | 60-69 | 5 | 5 | 8 | 2 | 6 | 5 | 6 | 4 | 9 | 11 | 8 | 9 | 9 | 11 | 12 | 11 | 14 |
|  | 70-79 | 5 | 4 | 3 | 4 | 4 | 6 | 7 | 4 | 2 | 3 | 3 | 3 | 6 | 3 | 6 | 2 | 3 |
|  | 80-89 | 3 | 5 | 3 | 3 | 1 | 0 | 0 | 1 | 2 | 3 | 1 | 2 | 3 | 2 | 1 | 4 | 1 |
|  | 90+ | 0 | 0 | 1 | 0 | 0 | 0 | 0 | 1 | 0 | 0 | 0 | 0 | 0 | 0 | 0 | 0 | 0 |
| **Legal status** | MHA | 33 | 66 | 109 | 129 | 220 | 222 | 168 | 178 | 175 | 214 | 159 | 228 | 286 | 292 | 219 | 244 | 220 |
|  | MHA 60b | 32 | 61 | 87 | 107 | 97 | 124 | 138 | 83 | 126 | 131 | 135 | 115 | 164 | 192 | 118 | 130 | 152 |
|  | MHA inf | 1 | 5 | 22 | 22 | 123 | 98 | 30 | 95 | 49 | 83 | 24 | 113 | 122 | 100 | 101 | 114 | 68 |
